# Association of Functional Limitations (ADLs) and Sexual Importance in Middle-Age and Older U.S. Adults.

**DOI:** 10.1093/geroni/igaf122.2324

**Published:** 2025-12-31

**Authors:** Lucas Prieto, Claire Leonard, Gilbert Gimm

**Affiliations:** George Mason University, Fairfax, Virginia, United States; George Mason University, Fairfax, Virginia, United States; George Mason University, Fairfax, Virginia, United States

## Abstract

Although prior studies have examined functional limitations (ADLs) in older adults, relatively few U.S. studies examine the association of disability and importance of sex in middle-age and older adults. Sexual health is a vital part of overall well-being in adults, therefore, understanding how older adults prioritize sex is essential. Given this gap, the goal of this study was to analyze a national sample of Wave 3 NSHAP participants and assess the relationship between disability as measured by the count of functional limitations (ADLs) and having any sexual importance among U.S. adults (50 years or older). Using respondent data from 4,371 NSHAP participants, we conducted bivariate and multivariate analyses of the likelihood of reporting sex as important among middle-age (50-64 years) and older U.S. adults (65 years or more). Nearly one-quarter (24.5%) of participants had any ADL limitations and a majority (55.5%) reported sex as important. Compared with middle aged adults (50-64 years), older adults (65+ years) had lower odds (OR = 0.30, p<.001) of reporting sex as important. Also, participants with 3 or more ADLs had lower odds of expressing sexual importance compared to zero ADLs (OR=.64, p=.005). Other factors that were significant with sexual importance included gender, education, race/ethnicity, relationship type, and health. These findings suggest that health care providers should include sexual health questions in their interactions with older adult patients regardless of sociodemographics. Results suggest the need to assess the link between functional limitations and sexual importance among middle-age and older U.S. adults to maintain well-being and healthy aging.

